# A novel nomogram based on HALP score for predicting time to glycemic stability in hospitalized type 2 diabetes patients

**DOI:** 10.3389/fendo.2026.1790538

**Published:** 2026-04-14

**Authors:** Ying Ke, Hongyuan Shi, Ming Zhang, Yi He, Fangyuan Liu, Xiaoyun Liu, Cuicui Lv

**Affiliations:** 1 Department of Urology, the Second Affiliated Hospital of Dalian Medical University, Dalian, Liaoning, China; 2 Department of Endocrine, the Second Affiliated Hospital of Dalian Medical University, Dalian, Liaoning, China; 3 Development Planning and Quality Management Department, the Second Affiliated Hospital of Dalian Medical University, Dalian, Liaoning, China; 4 Department of Discipline Construction and Research Management Department, the Second Affiliated Hospital of Dalian Medical University, Dalian, Liaoning, China

**Keywords:** glycemic stability, HALP score, nomogram, predictive mode, type 2 diabetes

## Abstract

**Background:**

Achieving rapid glycemic stabilization is a critical goal in the inpatient management of type 2 diabetes mellitus(T2DM). This study aimed to develop and validate a nomogram incorporating the hemoglobin, albumin, lymphocyte, and platelet (HALP) score and key clinical parameters to predict the time to glycemic stability in hospitalized T2DM patients.

**Methods:**

We conducted a retrospective analysis of 356 hospitalized T2DM patients. Baseline demographic, clinical, and laboratory data, including the HALP score, were collected. Univariate and multivariate Cox proportional hazards regression analyses were performed to identify independent predictors for the time to glycemic stability. The model’s discriminative ability was assessed using the concordance index, and its calibration was evaluated with calibration curves. Decision curve analysis (DCA) was used to estimate clinical utility.

**Results:**

Multivariate Cox regression analysis identified older age, lower hemoglobin level, higher hemoglobin A1c (HbA1c), and a lower HALP score as independent risk factors associated with a longer time to glycemic stability. These four variables were integrated into a prognostic nomogram, which demonstrated good predictive accuracy, with a C-index of 0.81(95% CI:0.78 – 0.84) in the training cohort. The calibration curves showed satisfactory agreement between predicted and observed probabilities. Decision curve analysis (DCA) indicated favorable clinical net benefit across a reasonable range of threshold probabilities.

**Conclusions:**

We developed and validated a practical nomogram that effectively predicts the time to glycemic stability in hospitalized T2DM patients, that may assist clinicians in early identification of patients at risk for delayed stabilization, thereby facilitating personalized management strategies and optimizing inpatient diabetes care.

## 1 Introduction

The management of hyperglycemia in hospitalized patients with type 2 diabetes mellitus (T2DM) remains a significant clinical challenge. Suboptimal glycemic control during hospitalization is strongly associated with increased risks of infection, prolonged length of stay, and higher morbidity (1, 2). To achieve rapid and stable glycemic targets, advanced therapeutic strategies combining continuous subcutaneous insulin infusion (insulin pump) with real-time continuous glucose monitoring (CGM) have been increasingly adopted in the inpatient setting (3). This integrated approach enables precise insulin delivery guided by real-time glucose trends, offering a promising method for overcoming glycemic instability. However, clinical experience reveals considerable individual variation in the time required to achieve glycemic stability with this intensive regimen. The ability to pre-emptively identify patients likely to experience a delayed response would facilitate personalized care, optimize resource allocation, and improve inpatient diabetes management outcomes.

Current prognostic assessments primarily rely on conventional parameters such as age, duration of diabetes, and hemoglobin A1c(HbA1c) levels (4). While valuable, these markers do not fully capture the patient’s underlying systemic inflammatory and nutritional status, which are critical determinants of metabolic homeostasis and treatment response (5, 6). Chronic low-grade inflammation, a hallmark of T2DM, exacerbates insulin resistance and complicates glycemic control (7). Concurrently, nutritional status, reflected by biomarkers like hemoglobin and albumin, influences physiological resilience. A composite index that integrates these dimensions could provide a more holistic prognostic tool.

The hemoglobin, albumin, lymphocyte, and platelet (HALP) score is a novel, integrative biomarker calculated from routine blood tests. It synthesizes markers of nutritional status (hemoglobin, albumin) and systemic inflammation (lymphocytes, platelets) into a single value, reflecting the balance between physiological reserve and inflammatory burden (8). This indicator was originally used in oncology research (9, 10) and played an important role in areas such as breast cancer and colon cancer, serving as an effective predictor of preoperative survival. In recent years, a lower HALP score has been associated with poorer outcomes in various chronic diseases, suggesting its role as a marker of compromised host status (11–14). In the context of T2DM, the inflammatory and nutritional pathways quantified by the HALP score are directly relevant to the pathophysiology of insulin resistance and the capacity for metabolic recovery (15–17). However, the prognostic utility of the HALP score for predicting short-term therapeutic outcomes in diabetes, particularly in patients undergoing intensive insulin pump and CGM therapy, has not been investigated.

Therefore, this study aimed to evaluate the HALP score as a novel predictor for the time to glycemic stability in hospitalized T2DM patients initiating combined insulin pump and CGM therapy. We hypothesized that a lower HALP score at admission, indicating a poorer inflammatory-nutritional state, would be independently associated with a longer time to achieve glycemic stability. Furthermore, we sought to develop and validate a practical nomogram incorporating the HALP score and other key clinical factors to provide an individualized risk assessment tool, thereby supporting clinical decision-making and personalized treatment planning in inpatient diabetes care.

## 2 Materials and methods

### 2.1 Patient recruitment and clinical variables collection

This retrospective analysis was conducted using data from consecutive hospitalized patients with T2DM who underwent combined insulin pump and CGM therapy between March 2024 and December 2025. All data were extracted from the hospital’s electronic medical record system. The inclusion criteria were as follows: (1) aged≥18 years, primary admission for glycemic optimization or uncontrolled T2DM; (2) initiation of combined insulin pump and CGM therapy. Exclusion criteria were: (1) presence of diabetic ketoacidosis or hyperosmolar hyperglycemic state at admission; (2) severe renal insufficiency (eGFR < 30 mL/min/1.73m²) or severe hepatic insufficiency; (3) active malignancy, systemic infection, or use of medications significantly affecting glucose metabolism (e.g., high-dose glucocorticoids); (4) CGM wear time of less than 72 hours or incomplete clinical/laboratory data.

Of the initial 382 patients screened, 26 were excluded due to missing outcome data (n = 12) or missing baseline covariate data (n = 14). The overall proportion of missing data for baseline clinical variables was low (< 3%). As this missingness fell below the commonly accepted threshold of 5-10%, we performed a complete case analysis without multiple imputation, which is unlikely to introduce significant bias or affect the robustness of the model estimates. A total of 356 patients were included in the final analysis.

The sample size was determined based on the events-per-variable principle for Cox regression analysis. With four predictors anticipated in the final model, a minimum of 40–60 events were required. Our study had >300 events, far exceeding this requirement. Additionally, using the formula proposed by Hsieh (18) et al. (two-sided α=0.05, power=0.80, expected HR for HALP score ≈2.0, event rate ≈70%, R²=0.2), the minimum required sample size was 290. Therefore, the included 356 patients met the sample size requirement and provided adequate statistical power for subsequent model development and validation. These patients were subsequently randomly divided into training (n=252) and validation (n=104) cohorts at a 7:3 ratio.

All patients underwent combined insulin pump and CGM therapy. For the purpose of this study, glycemic stability was defined as the achievement of Time in Range (TIR, glucose level 3.9–10.0 mmol/L) greater than 70% sustained for 24 consecutive hours, as determined by CGM data. The time to glycemic stability was calculated as the duration from the initiation of the combined therapy to the first occurrence of this 24-hour stability period. This definition was designed to exclude the influence of transient fluctuations and reflect sustained glycemic control. The time to glycemic stability was calculated as the duration from therapy initiation to the first occurrence of this 24-hour stability period. Therefore, in the Cox proportional hazards regression analysis, a hazard ratio greater than 1 indicates a longer time to achieve glycemic stability (i.e., a higher risk of delayed stabilization), while a hazard ratio less than 1 indicates a shorter time to achieve stability (i.e., faster stabilization).

The following baseline variables were collected within 24 hours of admission: age, sex, body mass index (BMI), HbA1c, and fasting plasma glucose. Complete blood count parameters, including hemoglobin, lymphocyte count, and platelet count, along with serum albumin, were obtained to calculate the HALP score using the formula: HALP = [Hemoglobin (g/L) × Albumin (g/L) × Lymphocyte count (×10^9^/L)]/[Platelet count (×10^9^/L)]. All patients provided written informed consent before participating in the procedure, which was approved by the ethics committee at Second Hospital of Dalian Medical University in accordance with the Declaration of Helsinki (approve number: 2023067).

### 2.2 Method for sample size determination

Based on the events-per-variable principle for Cox regression, with 4 predictors in the final model, at least 40–60 events were required. Our study had >300 events, far exceeding this. Additionally, using the Hsieh et al. (1991) formula (α=0.05, power=0.80, expected HR for HALP≈2.0, event rate≈70%, R²=0.2), the minimum required sample size was 290. We enrolled 356 patients, who were then randomly split into training (252) and validation (104) cohorts at a 7:3 ratio.

### 2.3 Establishment and validation of a nomogram for predicting time to glycemic stability

In this study, 356 patients were randomly assigned to a training cohort of 252 samples (70%) and an internal validation cohort of 104 samples (30%) using computer-generated random numbers. The randomization procedure was performed using the “sample” function in R software (version 4.2.3) with a fixed seed set to ensure reproducibility. Data on time to glycemic stability (event) and baseline clinical characteristics were integrated using the R package “survival”. The predictive significance of these characteristics was first assessed using univariate Cox regression analysis within the training cohort. Variables with a P-value < 0.05 in the univariate analysis were subsequently entered into a multivariate Cox proportional hazards regression model to identify independent prognostic factors. Based on the results of the multivariate analysis, a nomogram was constructed using the “rms” package in R to predict the probability of achieving glycemic stability at specific time points (e.g., 2^ed^, 4^th^, 6^th^ day after therapy initiation). The nomogram presents a graphical representation where each predictor is assigned a points score; the sum of these points corresponds to a total score from which the individualized probability of stability can be read.

During the validation phase, the total points for each patient in the validation cohort were calculated according to the established nomogram. The model’s discriminative ability was evaluated using the concordance index (C-index) and receiver operating characteristic (ROC) curve. The agreement between predicted probabilities and observed outcomes, was assessed using calibration plots. Furthermore, the clinical utility of the nomogram was quantified by decision curve analysis (DCA), which estimates the net benefit across a range of threshold probabilities.

### 2.4 Risk stratification based on the nomogram-derived score

The best risk score cut-off value for the nomogram-derived total risk score was determined for the entire dataset using the R package “maxstat” (Maximally selected rank statistics with multiple p-value approximations, version: 0.7-25). The minimum and maximum numbers of samples in each category were set at more than 25% and less than 75%, respectively. Patients were then stratified into high-risk and low-risk groups based on this cut-off value. The prognostic disparity in time to glycemic stability between these two groups was visualized using Kaplan-Meier survival curves generated by the survfit function in R. The statistical significance of the difference in survival distributions was assessed using the log-rank test.

### 2.5 Statistical analysis

The optimal cutoff values for continuous variables, including age, hemoglobin level, HbA1c, and the HALP score, were determined using X-tile software (version 3.6.1, Yale University). X-tile identifies cutoff points that maximize the difference in survival distributions (i.e., time to glycemic stability) based on the log-rank test statistic, thereby providing statistically optimal thresholds for risk stratification. For variables such as HbA1c, the software may generate multiple cutoffs to create clinically meaningful subgroups; the resulting cutoffs (e.g., 8.97% and 11.00%) were used for subsequent analyses. All statistical analyses were performed using SPSS software (version 24.0, IBM Corp., USA). The relevant hazard ratios (HRs) and 95% confidence intervals (CIs) were calculated using multivariate Cox regression analysis. For the DCA, the “ggDCA” R package was utilized. The area under the curve (AUC) was calculated by ROC analysis using the pROC package (version 1.17.0.1) of the R software (version 4.2.3). For all analyses, a two-tailed P-value of less than 0.05 was considered statistically significant.

## 3 Results

### 3.1 Baseline clinical characteristics

We conducted a retrospective analysis of the clinical data of hospitalized T2DM patients who received combined insulin pump and CGM therapy at our institution. A total of 356 patients who met the inclusion criteria and had complete follow-up information were enrolled and subsequently randomized into a training cohort (n=252) and a validation cohort (n=104).

According to our study definition (TIR >70% sustained for 24 hours), the proportion of patients achieving glycemic stability on the first day of therapy was 7.3%. This proportion increased to24.4%, 61.8%, and 86.8% by the 2^ed^, 4^th^, and 6^th^ day, respectively. Overall, the time to glycemic stability ranged from 1 to 11 days, with a median of 4 days.

Based on the optimal cutoff values determined by X-tile analysis (e.g., age ≥55 years, HbA1c ≥8.97%), the continuous variables in this study were transformed into categorical variables for analysis. The detailed baseline clinico-pathological characteristics of patients in the training and validation cohorts are summarized in **Table 1**.

**Table 1:** Representativeness of study participants.

Clinical characteristics		Entire cohort (N = 356)	Training cohort (N = 252)	Validation cohort (N = 104)
Age(years)	Median [min-max]	56 [20–88]		
	≤ 55	134 (37.64%)	89(35.32%)	45(43.27%)
	> 55	222 (62.36%)	163(64.68%)	59(56.73%)
Sex	Male	210 (58.99%)	151(59.92%)	59(56.73%)
	Female	146 (41.01%)	101(40.08%)	45(43.27%)
HbA1c (%)	Median [min-max]	9.93 [6.00 - 17.81]		
	< 8.97	116 (32.58%)	84(33.33%)	32(30.77%)
	8.97 - 11.00	126 (35.39%)	88(34.92%)	38(36.54%)
	> 11.00	114 (32.02%)	80(31.75%)	34(32.69%)
FPG (mmol/L)	Median [min-max]	8.17 [2.43 - 21.28]		
	≤ 10.64	253 (71.07%)	180(71.43%)	73(70.19%)
	> 10.64	103 (28.93%)	72(28.57%)	31(29.80%)
Hemoglobin (g/L)	Median [min-max]	142 [80 - 184]		
	≤ 140	159 (44.66%)	108(42.86%)	51(49.04%)
	> 140	197 (55.34%)	144(57.14%)	53(50.96%)
Platelet count (10^9^/L)	Median [min-max]	217 [87 - 785]		
	≤ 183	98 (27.53%)	69(27.38%)	29(27.88%)
	> 183	258 (72.47%)	183(72.61%)	75(72.12%)
Albumin (10^9^/L)	Median [min-max]	39.36 [23.87 - 74.23]		
	≤ 41.46	218 (61.24%)	152(60.32%)	66(63.46%)
	> 41.46	138 (38.76%)	100(39.68%)	38(36.54%)
Lymphocyte count	Median [min-max]	1.95 [0.23 - 6.94]		
	≤ 1.92	168 (47.19%)	124(49.21%)	44(42.31%)
	> 1.92	188 (52.81%)	128(50.79%)	60(57.69%)
HALP score	Median [min-max]	51.61 [2.96 - 240.73]		
	≤ 55.52	205 (57.58%)	145(57.54%)	60(57.69%)
	> 55.52	151 (42.42%)	107(42.46%)	44(42.31%)
BMI (kg/m²)	Median [min-max]	25.48 [16.94 -39.20]		
	≤ 29.61	314 (88.20%)	222(88.10%)	92(88.46%)
	> 29.61	42 (11.80%)	30(11.9%)	12(11.54%)

HbA1c, Hemoglobin A1c; FPG, Fasting Plasma Glucose; HALP, Hemoglobin, Albumin, Lymphocyte, and Platelet; BMI, Body Mass Index.

### 3.2 Construction of a combined Nomogram for individualized prediction

Univariate and multivariate Cox regression analyses were performed on clinical factors, including age, sex, BMI, hemoglobin, albumin, lymphocyte count, platelet count, the HALP score, HbA1c, and fasting plasma glucose (FPG). The results demonstrated that older age, lower hemoglobin level, higher HbA1c, and a lower HALP score were identified as independent risk factors for a longer time to glycemic stability (**Table 2**).

**Table 2:** Univariate and multivariate analyses of factors associated with glycemic stability.

Variable		Univariate analysis		Multivariate analysis	
		HR (95%CI)	*P* value	HR (95%CI)	*P* value
Age (years)	> 55 vs ≤ 55	1.77(1.415-2.206)	<0.001***	1.60(1.271-2.011)	<0.001***
Sex	Male vs ≤ Female	0.96(0.773-1.193)	0.716		
HbA1c (%)	< 8.97	–	–	–	–
	8.97 - 11.00	2.08(1.586-2.725)	<0.001***	2.02(1.524-2.684)	<0.001***
	> 11.00	1.50(1.153-1.950)	0.003**	1.36(1.035-1.786)	<0.027*
FPG (mmol/L)	> 10.64 vs ≤ 10.64	1.23(0.791-1.556)	0.087		
Hemoglobin (g/L)	> 140 vs ≤ 140	0.49(0.395-0.612)	<0.001***	0.60(0.470-0.757)	<0.001***
Platelet count (10^9^/L)	> 183 vs ≤ 183	0.76(0.599-0.965)	0.024**	0.85(0.663-1.093)	0.208
Albumin (10^9^/L)	> 41.46 vs ≤ 41.46	0.39(0.306-0.484)	<0.001***	0.86(0.630-1.183)	0.362
Lymphocyte count	> 1.92 vs ≤ 1.92	0.60(0.480-0.737)	<0.001***	0.85(0.669-1.079)	0.181
HALP score	> 55.52 vs ≤ 55.52	0.27(0.216-0.345)	<0.001***	0.39(0.271-0.547)	<0.001***
BMI (kg/m²)	> 29.61 vs ≤ 29.61	0.52(0.374-0.730)	<0.001***	0.80(0.570-1.132)	0.211

*P < 0.05, **P < 0.01, ***P < 0.001; HR >1 indicates longer time to glycemic stability (delayed stabilization); HR <no><1</no> indicates shorter time to stability (faster stabilization).

HbA1c, Hemoglobin A1c; FPG, Fasting Plasma Glucose; HALP, Hemoglobin, Albumin, Lymphocyte, and Platelet; BMI, Body Mass Index.

**Figure 1:**
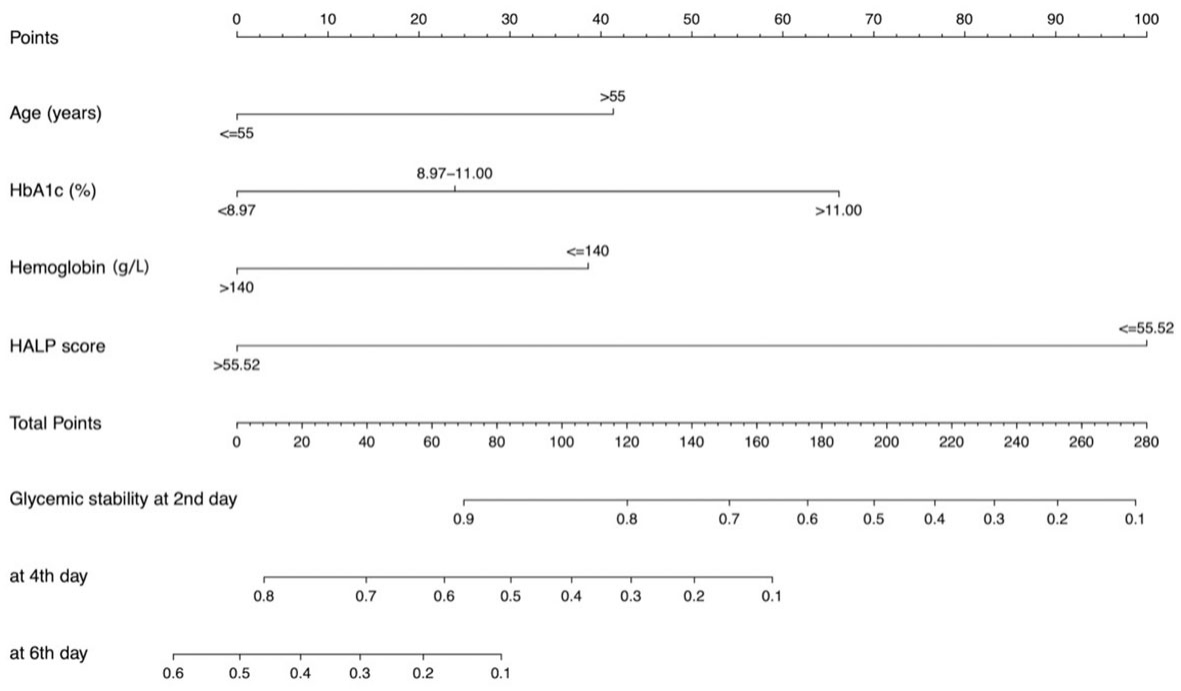
Development and validation of a predictive nomogram. Nomogram incorporating age, HbA1c, hemoglobin, and HALP score to estimate the probability of glycemic stability at 2^ed^, 4^th^, and 6^th^ day.

**Figure 2:**
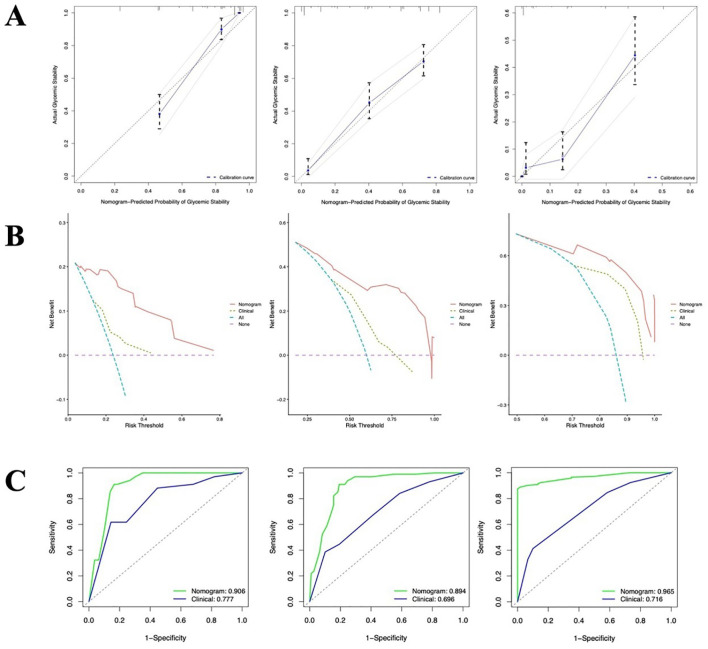
Validation of the nomogram in the training cohort. **(A)** Calibration curves showing the agreement between predicted probabilities and actual observed stability rates in the training cohort at 2^ed^, 4^th^, and 6^th^ day; **(B)** Time-dependent DCA comparing the net benefit of the nomogram with that of the clinical model at 2^ed^, 4^th^, and 6^th^ day; **(C)** ROC curves for predicting glycemic stability at 2^ed^, 4^th^, and 6^th^ day.

A nomogram integrating these four independent predictors was subsequently constructed based on the multivariate Cox regression analysis in the training cohort (**Figure 1**). The calibration plots indicated good agreement between the nomogram-predicted probabilities and the actual observed outcomes of achieving stability at 2^ed^, 4^th^, and 6^th^ day (**Figure 2A**). To illustrate the additive predictive value of the HALP score and hemoglobin level beyond conventional parameters, a baseline clinical model was built using only age and HbA1c. The C-index of the combined nomogram (0.81, 95% CI: 0.78-0.84) in the training set was notably higher than that of the baseline clinical model (0.66, 95% CI: 0.61-0.70), indicating superior discriminatory ability. DCA within a practical range of threshold probabilities further confirmed that the combined nomogram provided a greater net clinical benefit compared to the baseline model (**Figure 2B**). Consistently, the AUC values of the nomogram for predicting stability at each time point were all greater than those of the baseline clinical model in ROC analysis (**Figure 2C**).

### 3.3 Validation of predictive accuracy of the Nomogram

**Figure 3:**
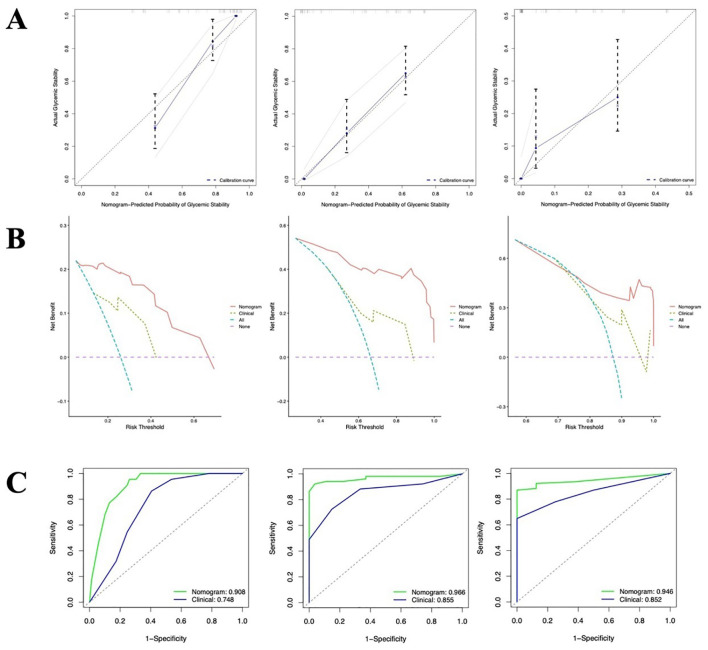
Validation of the nomogram in the validation cohort. **(A)** Calibration curves showing the agreement between predicted probabilities and actual observed stability rates in the validation cohort at 2^ed^, 4^th^, and 6^th^ day; **(B)** Time-dependent DCA comparing the net benefit of the nomogram with that of the clinical model at 2^ed^, 4^th^, and 6^th^ day; **(C)** ROC curves for predicting glycemic stability at at 2^ed^, 4^th^, and 6^th^ day.

In the validation cohort, the C-index of the nomogram for predicting the time to glycemic stability was 0.80(95% CI:0.76-0.84), which was higher than the C-index of the baseline clinical model(0.68, 95% CI:0.62-0.73). The calibration curve demonstrated good agreement between the predicted and observed probabilities of achieving stability at the 2^ed^, 4^th^, and 6^th^ day (**Figure 3A**). Furthermore, the DCA indicated that across a practical range of threshold probabilities, the nomogram provided a greater net clinical benefit than the “treat all” or “treat none” strategies, and consistently outperformed the baseline clinical model (**Figure 3B**). The ROC analysis showed that the AUCs of the nomogram for predicting stability at the 2^ed^, 4^th^, and 6^th^ day were 0.91、0.97 and 0.95 respectively (**Figure 3C**). These values were all superior to the corresponding AUCs of the baseline clinical model (0.75、0.86 and 0.85, respectively).

**Figure 4:**
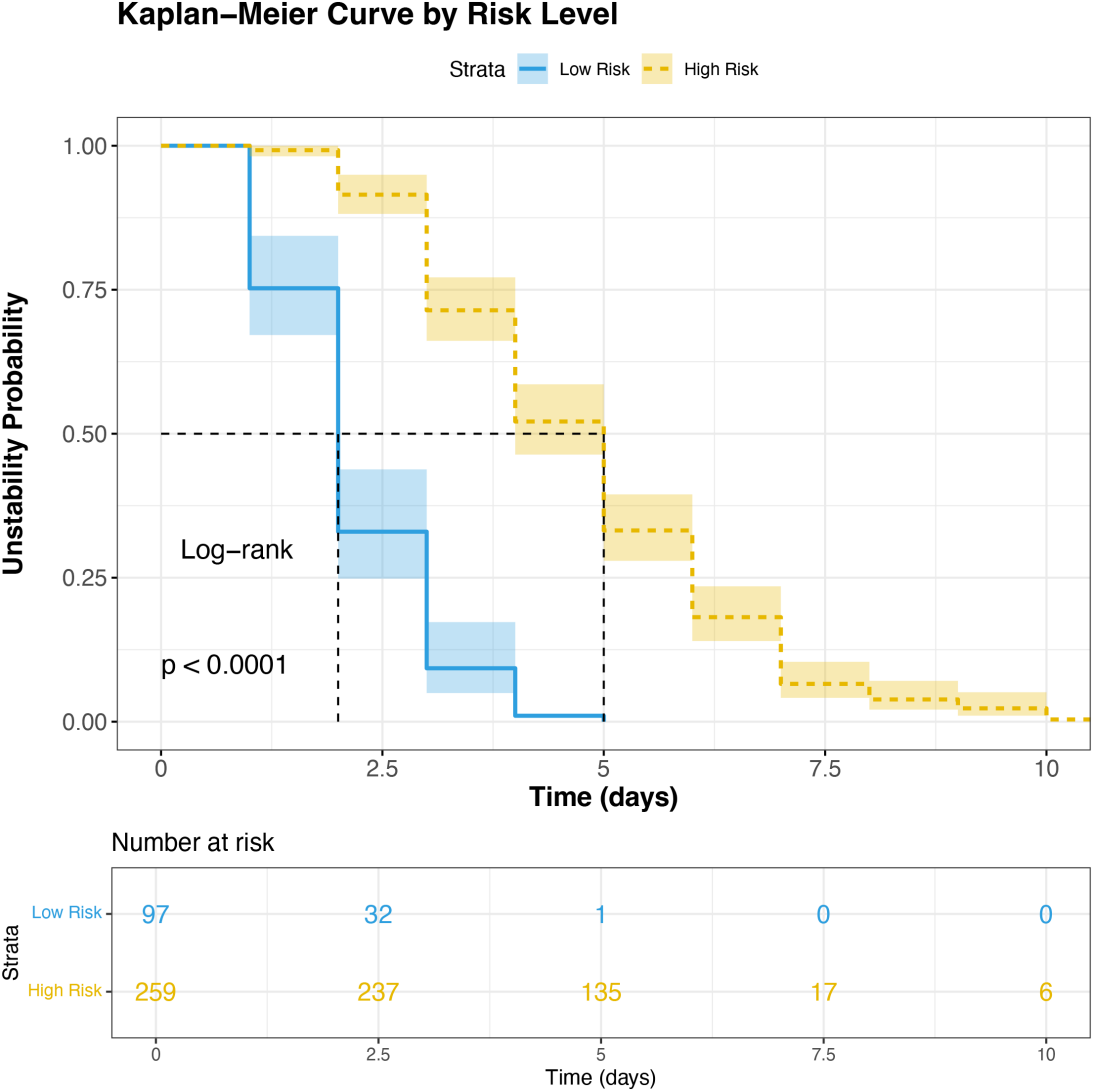
Predictive validation of the nomogram. Glycemic stability probability curves stratified by the nomogram-based risk score in the entire cohort.

Based on the nomogram modeling, all patients in this study were assigned a comprehensive risk score. The optimal cut-off value for this risk score was determined using the X-tile software. According to this cut-off, patients were stratified into high-risk and low-risk groups, with 72.8% (n = 259) in the high-risk group and 27.2% (n = 97) in the low-risk group. The Kaplan-Meier survival curves revealed a significant disparity in the time to glycemic stability between the two groups (Log-rank P < 0.001). The median time to stability was 5 days in the high-risk group, compared to only 2 days in the low-risk group (**Figure 4**).

## 4 Discussion

The primary finding of this study is the successful development and validation of a novel nomogram that integrates the HALP score with age, hemoglobin level, and HbA1c to effectively predict the time required for hospitalized T2DM patients to achieve glycemic stability (defined as TIR >70%) following the initiation of combined insulin pump and CGM therapy. With a robust C-index of 0.81, our model demonstrates not only the enduring prognostic value of established clinical metrics like HbA1c and age but also, for the first time, establishes the pre-treatment inflammatory-nutritional status, quantified by the HALP score, as a critical and independent determinant of a patient’s response to this advanced technological intervention.

The incorporation of age and HbA1c into the final model aligns with well-established pathophysiological principles. Older age is frequently associated with a constellation of factors detrimental to glycemic control, including increased insulin resistance, diminished beta-cell function, and a higher likelihood of polypharmacy and comorbidities, all of which can slow metabolic adaptation (19, 20). HbA1c serves as a reliable integrator of chronic glycemic exposure (21). A higher baseline HbA1c signifies a greater degree of established glucotoxicity and insulin resistance, representing a larger “metabolic debt” that must be repaid, thereby inherently prolonging the time course to stability even under intensive management (22, 23). The inclusion of hemoglobin level as an independent predictor underscores the role of physiological reserve. Anemia, indicated by lower hemoglobin, may compromise tissue oxygen delivery, potentially exacerbating insulin resistance in skeletal muscle and reducing overall metabolic capacity and resilience during the stress of rapid glycemic correction (24, 25).

The most significant and novel contribution of this research is the identification of the HALP score as a powerful independent prognosticator. This composite index moves beyond single-dimensional laboratory values to offer a holistic snapshot of the systemic milieu. A low HALP score synergistically indicates a state of poor nutritional support (reflected by low hemoglobin and albumin) coexisting with a dysregulated inflammatory environment (reflected by relative lymphopenia and/or thrombocytosis). Mechanistically, hypoalbuminemia reduces oncotic pressure and antioxidant capacity, while also serving as a marker of systemic inflammation and catabolism (26). Lymphopenia is increasingly recognized as a marker of nutritionally acquired immunodeficiency and systemic stress in chronic diseases, potentially indicating a weakened immunoregulatory response that is vital for metabolic homeostasis (27, 28). Conversely, thrombocytosis is a recognized component of the para-inflammatory state in diabetes and obesity; platelets are active inflammatory agents that can release cytokines promoting insulin resistance and endothelial dysfunction (29). Therefore, a patient presenting with a low HALP score likely possesses a diminished “host reserve” and an enhanced inflammatory burden. This state can fundamentally impair insulin signaling pathways, blunt tissue response to insulin, and reduce metabolic flexibility (30), explaining the observed protracted struggle to achieve glycemic stability despite receiving optimized, technology-enabled therapy.

The clinical utility of our nomogram extends beyond mere prediction to guide individualized management. Within 24 hours of admission, risk stratification can be performed using routine laboratory data: high-risk (e.g., older, anemic, with high HbA1c and low HALP score) have a median time to glycemic stability of up to 5 days, necessitating multidisciplinary intensive management (frequent monitoring and insulin adjustments by a specialized team, early nutritional intervention, close observation for complications, and management of hospitalization expectations); low-risk patients (median time 2 days) may be eligible for streamlined care and early discharge planning to optimize bed turnover. During hospitalization, the nomogram supports dynamic decision-making by predicting the probability of achieving stability on days 2, 4, and 6—if the probability of achieving the target by day 4 is below 50%, treatment can be intensified as early as days 2–3 (e.g., insulin optimization, combination therapy, or nutritional support) to shorten the time to target. In addition, the visualized probability curves facilitate doctor-patient communication, alleviating anxiety and improving adherence. Furthermore, the HALP score is derived from routine, low-cost blood tests enhancing the tool’s accessibility; its risk stratification results can also assist administrators in prioritizing bed allocation and staffing for high-risk patients, thereby optimizing both clinical decision-making and healthcare resource utilization.

Our study has several limitations that must be acknowledged. Firstly, although we have evaluated the stability of the multivariate Cox model based on its performance metrics, the model exhibits excellent discriminative ability (c-index of 0.81 in the training cohort and 0.80 in the validation cohort), well-calibrated predicted outcomes, and consistent internal validation. However, we acknowledge that formal collinearity diagnostics would enhance methodological rigor. We recognize this as a limitation and plan to incorporate VIF analysis in future external validation studies to further confirm the independence of these predictors. Secondly, the retrospective single-center design limits generalizability, and exclusion criteria (e.g., infection, renal impairment) restrict applicability to broader populations. The exclusion of patients with active infection, severe renal impairment (eGFR <30 mL/min/1.73m²), or glucocorticoid use was necessary to ensure a homogeneous cohort for model development, as these conditions independently influence both inflammatory status and glycemic control. However, these exclusion criteria also restrict the applicability of our nomogram to broader inpatient populations commonly encountered in clinical practice, such as those with concurrent infections or chronic kidney disease. Therefore, caution is warranted when extrapolating our findings to these patient subgroups, and external validation in more diverse populations is essential before widespread clinical implementation. Thirdly, the modest sample size and use of retrospective CGM (rather than real-time systems) warrant validation in larger, multicenter studies. Moreover, we used only random split-sample validation; future work should employ bootstrapping or cross-validation.

In addition, this study employed only random split-sample validation as the internal validation method, without conducting additional internal validation procedures such as bootstrap resampling or cross-validation. Although the consistency between the training and validation cohorts was satisfactory, future studies should incorporate these validation methods to further assess model stability. What’s more, our endpoint, while clinically meaningful, is one of several potential metrics; future studies could explore the model’s ability to predict other important outcomes like hypoglycemia events, cost of care, or long-term glycemic control post-discharge.

Finally, although we used categorized variables for clinical practicality, we acknowledge that this may reduce statistical efficiency; future studies with larger samples could evaluate the model using continuous forms of these predictors.

Nevertheless, we believe this study retains its value in several aspects. First, for appropriate patient populations where combined insulin pump and CGM therapy is applied as an advanced intensive glucose management strategy, our nomogram can provide precise support for clinical decision-making. Second, the core predictive factors identified in this study—HALP score, age, HbA1c, and hemoglobin—are all routine clinical indicators that can be obtained independently of CGM or insulin pump use. This implies that even in institutions without access to these technologies, the prognostic value of these risk factors themselves can still provide references for clinicians in assessing patient outcomes. We are currently constructing a prospective follow-up database and plan to address the following in future studies: (1) validate the correlation between nomogram-based risk stratification and total length of hospital stay; (2) assess the intervention effect of early identification of high-risk patients on hypoglycemia prevention; and (3) conduct health economic analyses to quantify the cost-effectiveness ratio of this tool.

## 5 Conclusion

We have developed and validated a practical, evidence-based nomogram that effectively predicts the time to glycemic stability in a challenging inpatient population. By integrating the novel HALP score—a biomarker of systemic inflammatory-nutritional status—with conventional clinical parameters, this tool provides a more comprehensive assessment of a patient’s likelihood for a rapid therapeutic response. It holds significant promise for enhancing clinical decision-making at the point of care, enabling early identification of vulnerable patients, and paving the way for more personalized, proactive, and efficient inpatient diabetes management. Future prospective studies are encouraged to validate its utility and explore its impact on hard clinical and economic outcomes.

## Data Availability

The raw data supporting the conclusions of this article will be made available by the authors, without undue reservation.
